# An evaluation of cervical maturity for Chinese women with labor induction by machine learning and ultrasound images

**DOI:** 10.1186/s12884-023-06023-4

**Published:** 2023-10-18

**Authors:** Yan-Song Liu, Shan Lu, Hong-Bo Wang, Zheng Hou, Chun-Yu Zhang, Yi-Wen Chong, Shuai Wang, Wen-Zhong Tang, Xiao-Lei Qu, Yan Zhang

**Affiliations:** 1 School of Computer Science and Engineering, Beihang University, Beijing, 100191 China; 2 Department of Obstetrics and Gynecology, Peking University Third Hospital, Beijing, 100191 China; 3 School of Instrumentation and Optoelectronic Engineering, Beihang University, Beijing, 100191 China

**Keywords:** Machine learning, Cervical maturity, Bishop score, Ultrasound, Labor time

## Abstract

**Background:**

To evaluate the improvement of evaluation accuracy of cervical maturity for Chinese women with labor induction by adding objective ultrasound data and machine learning models to the existing traditional Bishop method.

**Methods:**

The machine learning model was trained and tested using 101 sets of data from pregnant women who were examined and had their delivery in Peking University Third Hospital in between December 2019 and January 2021. The inputs of the model included cervical length, Bishop score, angle, age, induced labor time, measurement time (MT), measurement time to induced labor time (MTILT), method of induced labor, and primiparity/multiparity. The output of the model is the predicted time from induced labor to labor. Our experiments analyzed the effectiveness of three machine learning models: XGBoost, CatBoost and RF(Random forest). we consider the root-mean-squared error (RMSE) and the mean absolute error (MAE) as the criterion to evaluate the accuracy of the model. Difference was compared using t-test on RMSE between the machine learning model and the traditional Bishop score.

**Results:**

The mean absolute error of the prediction result of Bishop scoring method was 19.45 h, and the RMSE was 24.56 h. The prediction error of machine learning model was lower than the Bishop score method. Among the three machine learning models, the MAE of the model with the best prediction effect was 13.49 h and the RMSE was 16.98 h. After selection of feature the prediction accuracy of the XGBoost and RF was slightly improved. After feature selection and artificially removing the Bishop score, the prediction accuracy of the three models decreased slightly. The best model was XGBoost (*p* = 0.0017). The *p*-value of the other two models was < 0.01.

**Conclusion:**

In the evaluation of cervical maturity, the results of machine learning method are more objective and significantly accurate compared with the traditional Bishop scoring method. The machine learning method is a better predictor of cervical maturity than the traditional Bishop method.

## Contribution

- What does this work add to what is already known?

Traditionally cervical maturity was evaluated by the Bishop scoring method with sub-optimal accuracy. In this study, cervical maturity is evaluated by adding objective clinical data and ultrasonic data using machine learning model to improve the evaluation accuracy. The results of the model showed that induction of labor time (ILT), measurement time (MT), and whether amniotomy performed in the mode of induced labor have an important impact on the time from induction of labor to labor (ILTLT).

- What are the clinical implications of this work?

At present, Bishop score is used to evaluate the cervical maturity in clinical setting, but the score is greatly affected by subjective factors. Hence, we consider introducing the objective information collected from ultrasound images and combining that with the machine learning to predict the cervical maturity objectively and accurately.

## Background

Cervical maturity refers to softening, shortening, disappearance, and expansion of the cervix before delivery. It is an important factor to judge the timing of delivery. It can effectively prevent emergencies and plays an important role in the success of induced labor [1, 2]. Bishop score is often used as the standard to evaluate the cervical maturity in clinical settings [3]. While evaluating the cervical maturity by the traditional Bishop scoring method, doctors have measured the five indicators of pregnant women—dilatation, effacement, station, consistency, and position—through clinical examination, and then used the Bishop score to evaluate the cervical maturity. The scoring process depends on doctors’ instruction [4]. However, the scoring results are to a larger extent subjectivity and relying on individual experience of the doctors, which impacts the accuracy of cervical maturity assessment [5]. Cervical maturity assessment lacks accurate biological parameters.

In recent years, ultrasound technology has gradually expanded [6], and it has also made some progress in cervical maturity evaluation. The technology of measuring cervical length by transabdominal ultrasound, transperineal ultrasound, and transvaginal ultrasound has been recognized by more experts [7–10]. Owing to the advantages of noninvasive, convenient, and low cost [11], the application of cervical ultrasonography is becoming more general in clinical practice. For example, Berghella et al. used cervical ultrasonography to prevent preterm birth [12]; Friedman used transabdominal ultrasound to screen the women with short cervix [13]. Compared with transabdominal ultrasound, transvaginal ultrasound can provide more information for its higher accuracy and better image quality [7]. Therefore, our study used transvaginal ultrasound to obtain the objective data such as cervical length and cervical opening angle.

With the theory and technology of machine learning development, the machine learning method based on statistical probability is outstanding in the classification and regression tasks in many datasets [14, 15]. In recent years, machine learning technology has found wider application in medical diagnosis and prediction [16, 17], such as prediction of shoulder dystocia [18], postpartum hemorrhage [19] and postpartum depression [20]. However, there is no consistent standard for how to use some objective biological parameters to predict the cervical maturity [21].

In this study, we investigated a more objective parameter to evaluate the cervical maturity and proposed using the time from induced labor to labor as the parameter: the shorter the time from induced labor to labor, the higher the cervical maturity, and vise versa. We used the collected clinical and ultrasound data to train the machine learning models which was used for prediction of the time from induced labor to labor. In addition, STROBE [22] and TRIPOD [23] were complied with in this study.

## Methods

### Data preparation

Data of pregnant women who were examined and had their delivery in Peking University Third Hospital between December 2019and -January 2021 were collected. The study population was Chinese women.

The inclusion criteria were full-term pregnant women who had indications of induced labor but no contraindications to vaginal delivery and could tolerate vaginal delivery. Bishop score of ≤ 6; pregnant women aged 18–50 years (both ends not included) with Bishop score of ≤ 6; head position of single live fetus was to be indicated by prenatal ultrasound examination and the size of fetus to be consistent with the gestational week, and *non-stress Test* (NST) reactive type as indicated by fetal heart rate monitoring. All data were confirmed by the puerperal woman, and signed informed consent was obtained.

**Fig. 1:**
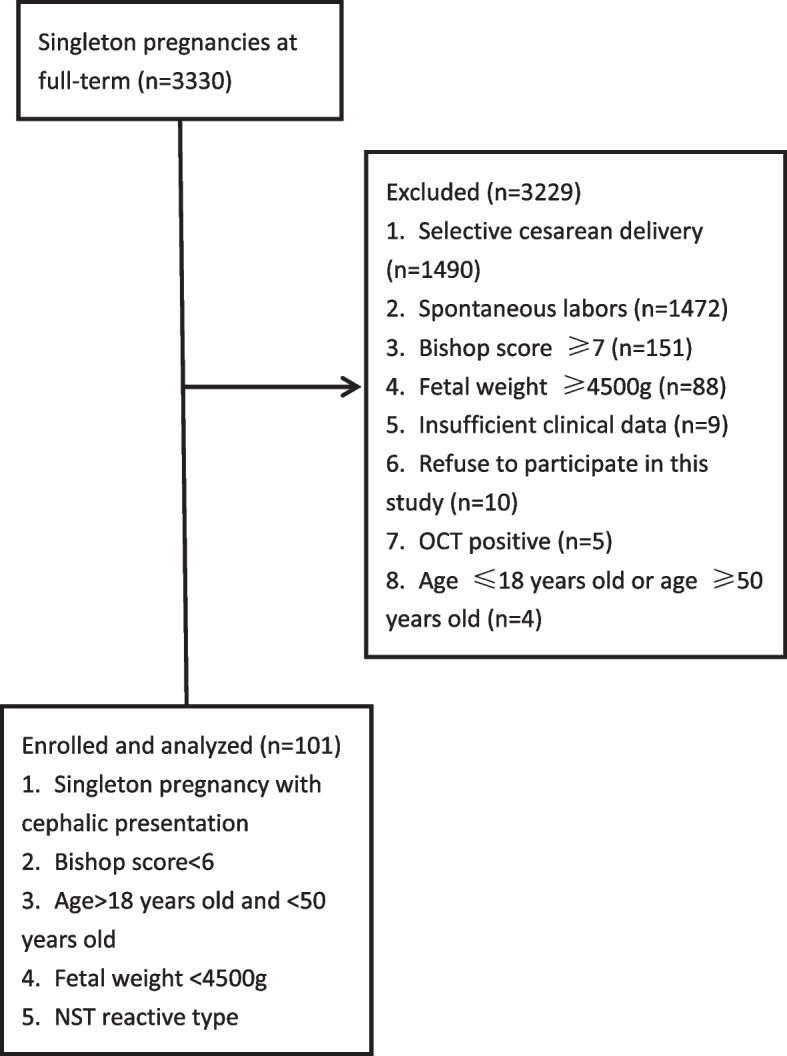
Flow diagram for inclusion of study population

Patients with cervical Bishop score of > 6, patients aged ≤ 18 years or ≥ 50 years, and patients who did not agree to participate in this study either by themselves or because of their families were excluded. A flow diagram for the population selection is shown in Fig. 1.

**Fig. 2:**
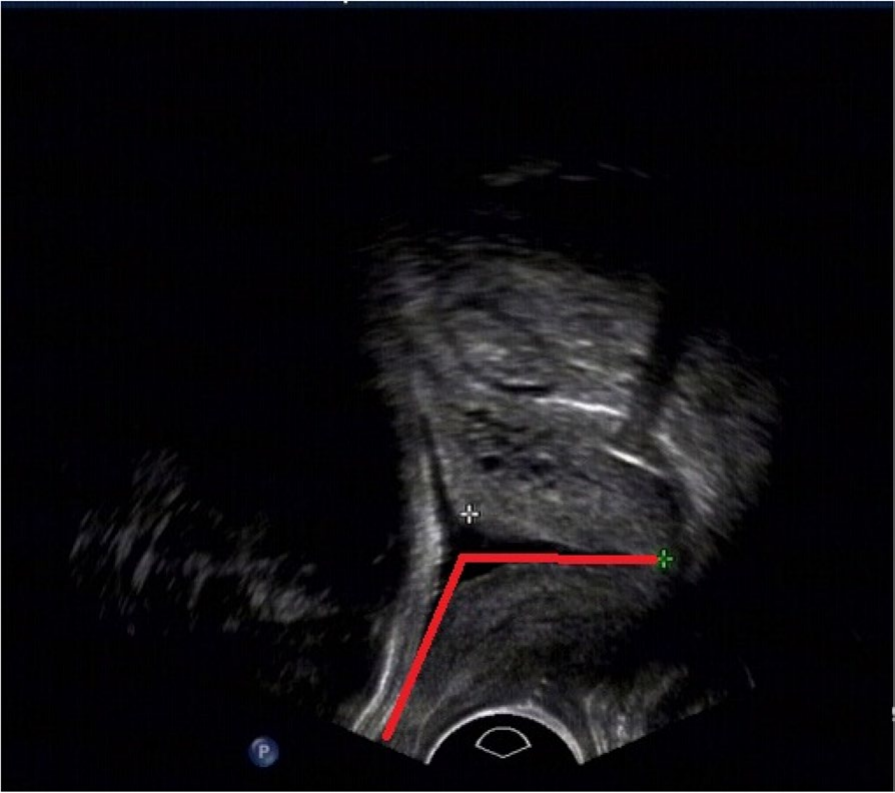
Illustration of the feature of angle

For each puerperal woman, we collected data by the following parameters: age, primiparity/multiparity, cervical length, angle, induced labor time (ILT), measurement time (MT), the time from measurement to induced labor (MTLILT), Bishop score, method of induced labor, and the time from induced labor to labor (ILTLT). From ultrasound scanning, cervical length and angle were determined. Ultrasound images were obtained by standardized transvaginal ultrasound with a frequency of 5.0–9.5 MHz. The cervical length was measured in centimeter. The angle was measured in radian. Angle refers to the included angle of the uterine wall at the cervical opening, which is shown by the two red lines in Fig. 2.

Owing to the lack of data and inconsistent format of the original data, data cleaning and preprocessing are needed. After eliminating the missing data, the data of 101 puerperal women were available for evaluation. The subjects of our study were all Chinese Han pregnant women. All pregnant women had successful labor. The proportion of vaginal delivery was 87.13% (88 women), the proportion of caesarean delivery was 12.87% (13 women), and the proportion of multiparity was 20.79%. Three women delivered with caesarean delivery because cervical dilation arrested for 6 h at 4 cm, and other 10 caesarean deliveries because of active phase arrest. Some basic biological characteristics of 101 pregnant women participating in the research are shown in Table 1.

**Table 1:** Characteristic statistics of pregnant women

	Cervical length(cm)	age	Angle(radian)	Bishop score
Lower Quartile	1.76	30.00	1.58	4.00
Median	2.4	32.00	1.79	5.00
Upper Quartile	2.89	35.00	2.06	6.00
	ILTLT (hour)	ILT (week)	MT (week)	BMI
Lower Quartile	15.00	41.73	41.64	19.96
Median	26.00	41.91	41.86	22.22
Upper Quartile	47.00	42.76	42.71	23.92

*ILT* Induction of labor time, *MT* Measurement time, *ILTLT* The time from induction of labor to labor

### Data preprocessing

Bishop scoring method was used as the control group of this study. To evaluate the prediction ability of the control group and make the traditional Bishop evaluation method comparable with the machine learning method proposed by us, we processed the data of the control group as follows (taking a group with Bishop score of 6 as an example): The root mean square error (RMSE) was calculated from the real value of the time from induced labor to labor for each patient in this group and then the average value was taken. Then, in this group, a total of 31 mean square error values can be obtained. The groups with Bishop scores of 0, 1, 2, 3, 4, 6, and 7 could generate a total of 101 RMSE values similar to the procedure mentioned above. This processing method, which takes the mean value from induced labor to labor of each Bishop score group as the predicted value of each group, is the most fair data processing method of the control group. In addition, we also conducted experiments to fit bishop score and the time from induction of labor to labor with linear regression model.

**Fig. 3:**
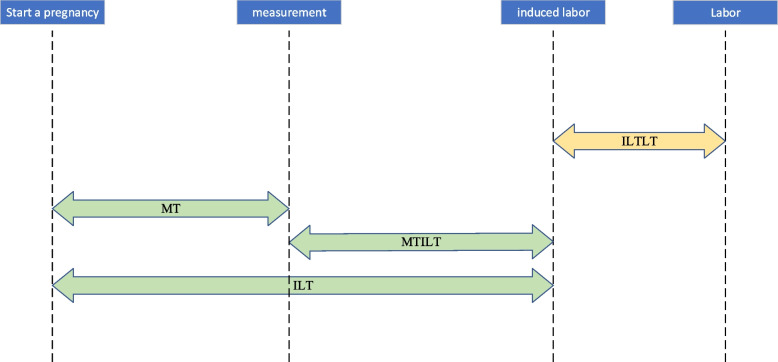
Diagram of four time variables; *ILTLT* The time from induction of labor to labor, *MT* Measurement time, *MTILT* Measurement time to induced labor time, *ILT* Induction of labor time

We finally used cervical length, Bishop score, angle, age, induced labor time (ILT), measurement time (MT), the time from measurement to induced labor (MTILT), method of induced labor, and primiparity/multiparity as the input of three machine learning algorithms in the experimental group. The output of machine learning algorithm is the predicted time from induced labor to labor. The explanation of each feature is described in Table 2. Among them, the relationship between the three time variables is shown in Fig. 3. In Fig. 3, the three variables below in green are the input variables of the model, while the one above in yellow is the result that the model aims to predict, which is the output variable of the model. The induced labor time and measurement time is expressed in weeks, and the time from measurement to induced labor is expressed in days.

**Table 2:** Feature introduction

Feature name	Feature interpretation	Source
Cervical length	The cervical length of puerperal woman	Ultrasonic data
Bishop score	The score of Bishop method	Clinical data
Angle	Angle of the uterine wall at the cervical opening	Ultrasonic data
Age	Age of the pregnant woman	Clinical data
Induced labor time (ILT)	Induced labor time refers to the time when the doctor induces labor for pregnant women, and the unit is converted to weeks	Clinical data
Measurement time (MT)	The measurement time is the time when the doctor carries out ultrasonography on pregnant women, and the unit is converted to weeks	Clinical data
The time from measurement to induced labor (MTILT)	The time from measurement to induced labor is the time interval from ultrasonography to induction of labor, and the unit is converted into days	Clinical data
Method of induced labor	Methods of induced labor adopted by pregnant women	Clinical data
Primiparity/multiparity	Is primiparity or multiparity	Clinical data

Method of induced labor and primiparity/multiparity are category features, which cannot be directly used as the input of the machine learning model. These two features need to be processed further. The categorical features are not numerical features but discrete sets, such as method of induced labor, that include misoprostol, oxytocin, amniotomy, Propess (PGE2), or none. When dealing with the two category features of primiparity/multiparity and method of induced labor, we used one-hot coding method to convert them into numerical characteristics. The specific process is to use two values to indicate whether the puerperal woman had a primiparity or multiparity delivery. If the value is 10, it means primiparity, and if the value is 01, it means multiparity. Five numerical values were used to represent the methods of induced labor of pregnant women. We selected three real pregnant women in the data set and showed the one-hot coding of their method of induced labor as presented in Table 3. The first Puerperal woman used one method to induce labor: oxytocin. The second Puerperal woman was induced by two methods: oxytocin and miso. The third woman was induced by three methods: misoprostol, oxytocin and amniotomy.

**Table 3:** One-hot coding method to convert category features into numerical value. Example explanation of one-hot coding

Methods	Misoprostol	Oxytocin	Amniotomy	Propess	None
Puerperal woman 1: oxytocin	0	1	0	0	0
Puerperal woman 2: misoprostol and oxytocin	1	1	0	0	0
Puerperal woman 3: misoprostol、oxytocin and amniotomy	1	1	1	0	0

“Methods” represent the methods of labor induction. Puerperal woman 1 only used “oxytocin”. Puerperal woman 2 used “misoprostol” and “oxytocin”. Puerperal woman 3 used “misoprostol”、 “oxytocin” and “amniotomy”

### Machine learning algorithm

**Fig. 4:**
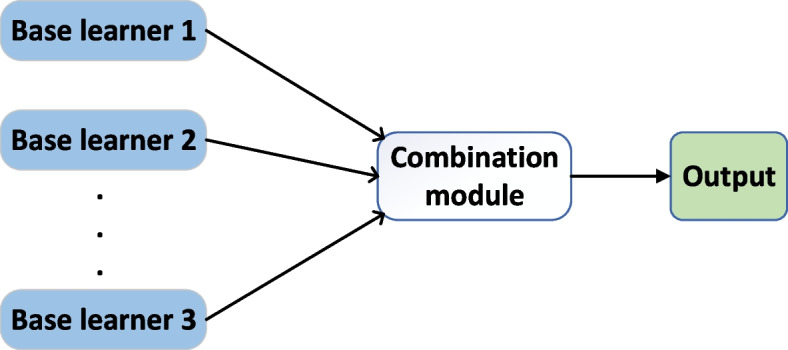
Overview of Ensemble learning

Ensemble learning is considered to be the most advanced solution to solve machine learning problems (Fig. 4). In recent years, a large number of ensemble learning models have been created and that showed strong learning performance [24]. By combining multiple learners, ensemble learning can usually obtain significantly superior generalization performance than a single learner.

Therefore, we used the three ensemble learning models, that are commonly used and perform well in most cases to predict the cervical maturity. They are XGBoost (eXtreme Gradient Boosting) [25], CatBoost (an implementation of Gradient Boosted Decision Trees) [26], and Random forest (RF) [27]. Among them, XGBoost and CatBoost models are serialization methods, whereas RF model is parallelization method.

XGBoost, CatBoost, and RF are often used for regression tasks, but they are different from each other. RF is a more advanced algorithm based on decision tree. RF is a forest constructed in a random way, and this forest is composed of many unrelated decision trees. Its working principle is to generate multiple decision trees to learn and predict independently. The predictions generated by these decision trees are finally combined into a single prediction, so they are better than any single base learner. XGBoost has a higher precision and flexibility. It supports not only Classification and Regression Tree (CART, a sort of decision tree) but also linear classifier. XGBoost also adds a regularization term to control the complexity of the model. It draws lessons from the practice of RF and supports column sampling, which not only reduces over fitting but also reduces calculation. CatBoost loss function is the same as the XGBoost loss function. The specialty of CatBoost is to deal with category feature efficiently and reasonably. If a category feature has low-cardinality features, that is, the number of set elements formed by the de-duplication of all values of the feature is relatively small, then the advantage of CatBoost cannot be brought into play. In this case, we generally use the one-hot coding method to convert the category feature into numerical type, similar to the processing of the two category features of primiparity/multiparity and method of induced labor introduced in the data preprocessing section. In addition, CatBoost also solves the problems of gradient bias and prediction shift, hence, reduces the occurrence of over fitting and improves the accuracy and generalization ability of the algorithm.

### Feature importance

In machine learning tasks, it is usually necessary to make further feature selection, that is, select the features that have a great impact on the prediction results. Then, train the model again with the selected features. Feature selection eliminates some data that have a little impact on the results, which helps to alleviate the problem of less data and improve the accuracy. In addition, feature selection can also reduce the computational overhead and the time of model training. For the three machine learning methods of XGBoost, CatBoost, and RF, we analyze the importance of each feature in different models and make feature selection. For the method of feature selection, sum the value of feature importance, and then calculate the proportion for each feature. The features with a proportion of < 0.033 are discarded, and the remaining features are used as the input of the machine learning model to re-train the model.

We use the methods embedded in these three machine learning models to estimate the importance of features. If a feature is particularly helpful to improve the accuracy of the model, it is considered important. In the model we use, the importance of features is measured by Gini index. The calculation formula of Gini index is

1 $$\begin{array}{c}G{I}_{m}=\sum\limits_{k=1}^{\left|K\right|}\sum\limits_{k\ne {k}^{\prime}}{p}_{mk}{p}_{{mk}^{\prime}}=1-\sum\limits_{k=1}^{\left|K\right|}{p}_{mk}^{2}\end{array}$$

where, K means there are K categories,${p}_{mk}$ represents the proportion of category k in node m. The importance of feature j in node m is the variation of Gini index before and after node m splitting.

### Modelling

Owing to the small amount of data collected, to ensure the higher credibility of the results, we have conducted a five-fold cross-validation for each machine learning method. In each fold, 80% of the data are used as the training set, and 20% of the data are used as the test set. 80%/20% segmentation is also commonly used in machine learning algorithm that deals with medium or small samples. This ratio helps to ensure that there are enough training samples to build a robust model and enough test samples to evaluate that model.

We adjusted the following parameters to make the performance of several machine learning models better. The number of base learners in XGBoost is set to 40. In general, the more the number of spanning trees, the more accurate the model is, but has longer model training. Learning_rate is a parameter used to control the step size of each gradient descent during the model training, which is set to 0.1; max_depth is the maximum depth of each tree, which can be used to prevent over fitting, and that is set to 3. In CatBoost, the number of base learners is set to 70, and the loss function is specified as the RMSE. The number of base learners in the RF is set to 165, and the minimum sample number of leaf nodes is set to 6. When the CART tree of the base learner is divided, the evaluation standard of the feature is set as Gini index.

### Evaluation

To evaluate the accuracy of the model, we use RMSE and MAE The calculation formula is shown in formulas (2) and (3), where ${y}_{i}$ is the true value and $\widehat{{y}_{i}}$ is the predicted value.

2 $$\begin{array}{c}RMSE=\sqrt{\frac{1}{m}\sum\limits_{i=1}^{m}{\left({y}_{i}-\widehat{{y}_{i}}\right)}^{2}}\end{array}$$

3 $$\begin{array}{c}MAE=\frac{1}{m}\sum\limits_{i=1}^{m}\left|{y}_{i}-\widehat{{y}_{i}}\right|\end{array}$$

The data of the control group were obtained as per the method described in the section “Data preprocessing.” In the experimental group, 101 RMSE values can also be obtained by making the RMSE between the predicted value and the real value of the model. The 101 RMSE values of the control group and the 101 RMSE values of the experimental group were tested by t-test, and the *p*-value was calculated.

The *p*-value of model significance test is calculated by stats.ttest_ind() in Python’s standard scientific calculation library SciPy. If *p* < 0.05, it is considered to be statistically significant difference; if *p* < 0.01, it is considered to be prominent statistically significant difference; if *p* < 0.001, it is considered to be very prominent statistically significant difference.

## Results

### Traditional Bishop scoring method

As described in the “Methods” section, we gave the prediction value of the time from induced labor to labor by the traditional Bishop scoring method. The processing method of taking the mean value as the predicted value is fair to minimize the prediction error. The prediction results are detailed in Table 4. The table presents the sample number, predicted value, MAE of each group, RMSE of each group, maximum time difference of each group, overall MAE, and overall RMSE. MAE and RMSE were calculated using the formulas described above. The predicted value and the maximum time difference of each group were measured in hours. The results showed that MAE and RMSE decreased with the increase of Bishop score. This also shows that Bishop score has some value as a standard of cervical maturity.

**Table 4:** Experimental results of traditional Bishop scoring method

Bishop score	0	1	2	3	4	5	6	7
Sample numbers	0	0	0	3	25	42	31	0
Prediction value	Null	Null	Null	50.33	43.76	30.88	26.95	Null
MAE	Null	Null	Null	35.11	21.93	18.80	16.81	Null
RMSE	Null	Null	Null	37.81	25.27	24.84	21.76	Null
maximum time difference	Null	Null	Null	87.00	88.50	120.00	97.50	Null
MAE	19.45							
RMSE	24.55							

*MAE* Mean Absolute Error, *RMSE* Root mean square error

At the same time, the experimental results shown in Table 4 also exposed the limitations of Bishop score. Taken the group with a Bishop score of 5 as an example: the number of samples is 42, and the predicted value is 30.88, but the MAE of this group is 18.80, the RMSE is 24.84, and the maximum time difference is 120. This shows that although Bishop score has some significance for cervical maturity evaluation on the whole, there are great differences among different pregnant women with the same Bishop score. The overall MAE of Bishop scoring method is 19.45, and the overall RMSE is 24.55, indicating that Bishop scoring system has low accuracy and large deviation in evaluating cervical maturity. The MAE and RMSE results of the bishop scoring method that obtained by linear regression model in Table 5. The results in Table 5 showed that MAE and RMSE of linear regression prediction results are 23.17 and 29.60. It is worse than our proposed method, which takes the mean ILTLT of each bishop score group as the predictive value.

**Table 5:** Accuracy of all models

	Training set MAE	Training set RMSE	Test set MAE	Test set RMSE	*p*-value
Before feature selection					
XGBoost	8.26	10.64	13.68	17.16	0.0023
CatBoost	9.37	11.68	13.92	17.45	0.0039
RF	11.58	15.18	14.21	18.05	0.0071
After feature selection					
XGBoost	8.41	10.80	13.49	16.98	0.0017
CatBoost	9.61	11.83	13.74	17.25	0.0028
RF	11.00	14.46	14.10	17.86	0.0057
After eliminating Bishop score					
XGBoost	8.35	10.84	13.82	17.31	0.0032
CatBoost	9.69	11.98	13.82	17.40	0.0036
RF	10.81	14.32	14.11	17.83	0.0058
Only Bishop score to predict ILTLT					
Polynomial regression	21.01	27.34	23.17	29.60	

*MAE* Mean Absolute Error, *RMSE* Root mean square error

### Machine learning method

After inputting all features into three machine models, the prediction results are presented in Table 5. The MAE and the RMSE in the training set and the MAE and the RMSE in the test set of the three models are closely matching, indicating that the model has not been over fitted. Among them, the XGBoost model performs the best with the MAE of 13.68 and the RMSE of 17.16. The MAE of the control group was 19.45, and the RMSE was 24.55. Our machine learning model has improved by 5.77 and 7.39, respectively, in two error indicators for evaluating prediction accuracy. Even the worst performing RF model has an improvement of 5.24 and 6.5, respectively. The *p*-values of the three machine learning models are 0.0023, 0.0039, and 0.0071, respectively, which are < 0.01 with a significant statistical difference. It shows that the three machine learning models are significantly better than the traditional Bishop scoring method.

**Fig. 5:**
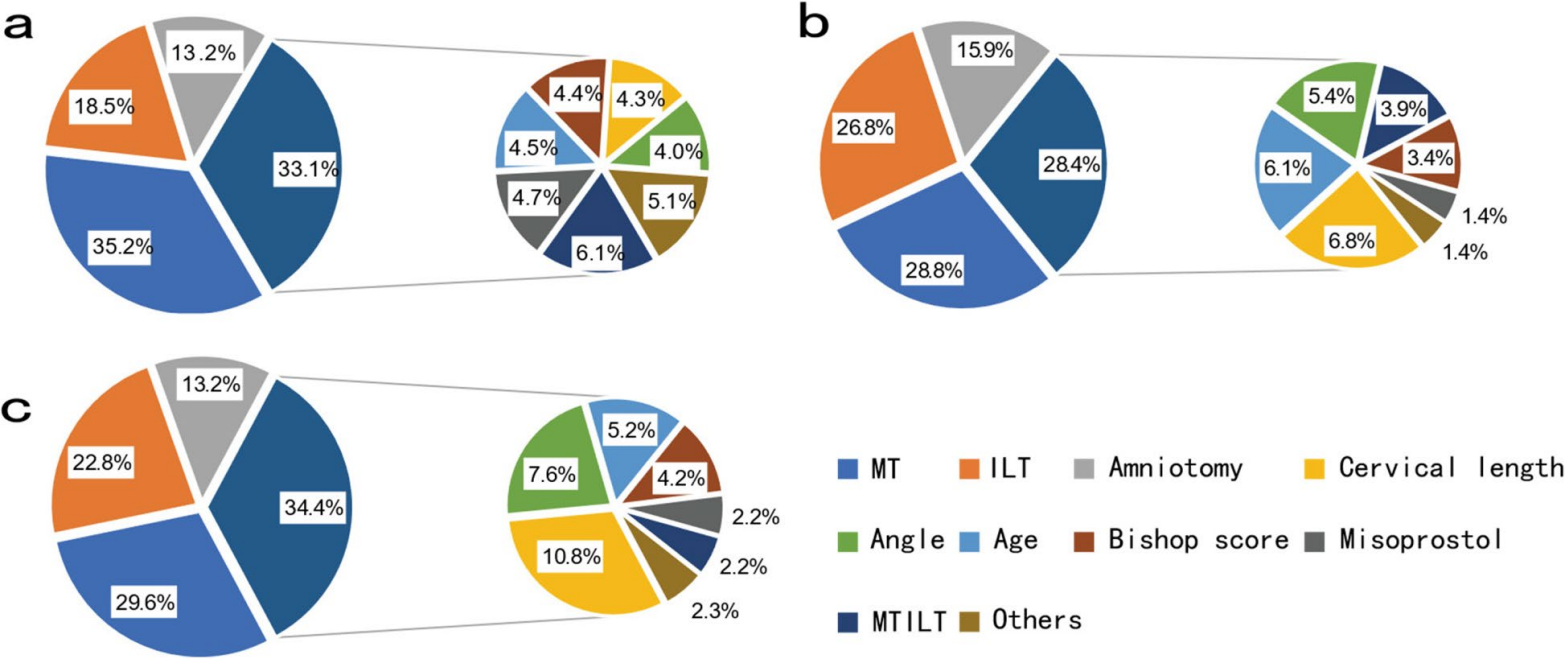
Key features of three models; **a**, XGBoost; **b**, Catboost **c**, Random Forest (RF); MTILT: time from measurement to induced labor. MT: time of measurement. ILT: time of induced labor. Misoprostol and amniotomy are two items under the method of induced labor. ‘Others’ is a collection of other unimportant features, including primiparity, multiparity, oxytocin, Propess, and none under category features

Figure 5 shows the importance of each feature in the XGBoost model (a), CatBoost model (b) and RF model (c). The feature importance of the output of the three models shows that the cervical length, Bishop score, angle, age, ILT, measurement time (MT), MTILT, and amniotomy or misoprostol that are used in the method of induced labor have an important impact on the accuracy of the model. The importance of ILT, MT, and amniotomy used in the method of induced labor is much greater than that of cervical length, Bishop score, angle, age, MTILT, and misoprostol that are used in the method of induced labor.

### After feature selection

Method introduced in the previous section was used to evaluate the importance of each feature and make feature selection. After feature selection, the remaining features of XGBoost are cervical length, Bishop score, angle, age, ILT, MT, MTILT, misoprostol, and amniotomy; the remaining features of CatBoost are cervical length, Bishop score, angle, age, ILT, MT, MTILT, and amniotomy; and the remaining features of RF selection are cervical length, Bishop score, angle, age, ILT, MT, and amniotomy.

The results of each model after feature selection are presented in Table 5. The results showed that after feature selection, the *p*-values of the three models are still < 0.01, indicating that the model is still significantly better than the traditional Bishop scoring method. Moreover, the prediction accuracy of the three models after feature selection is slightly improved.

### Machine learning method with Bishop eliminating

During feature selection, the feature of Bishop score was selected. In the XGBoost model, the feature of Bishop score ranked 7th in importance. In the CatBoost model, the feature of Bishop score ranked 8th in importance. In the RF model, the feature of Bishop score ranked 7th in importance. After removing the Bishop score, we re-train the model, and the results are presented in Table 5. After the Bishop score is removed, the three models become less significant (*p*-values increased). The MAE of XGBoost increased from 13.49 h to 13.82 h, with an increase of 2.4%; the RMSE increased from 16.98 h to 17.31 h, with an increase of 1.9%. The MAE of CatBoost increased from 13.74 h to 13.82 h, with an increase of 0.5%; the RMSE increased from 17.25 h to 17.40 h, with an increase of 0.8%. The prediction accuracy of RF (i.e., MAE and RMSE) did not change significantly. The experimental results in Table 5 show that the Bishop score also has some contribution to the accuracy of the model, but its contribution is limited. The machine learning model /excluding Bishop score is still significantly better than the traditional simple Bishop score system.

## Discussion

This study is a retrospective clinical research. The definition of labor failure induction was the inability to achieve the active phase. But the definition of the active phase has always been controversial. The standard for defining the active phase is based on cervical dilation > 4-5 cm. In our center, we use 3 cm as standard for defining the active phase traditionally. Thus in this study all 101 pregnant women achieved the active phase and the rate of successful labor induction was 100%. But among them 3 women delivered with caesarean delivery because cervical dilation arrest last for 6 h at 4 cm. So according to the recent standard the rate of successful labor induction was 97.03% in this study.

Cervical maturity is a necessary condition for successful induction, but there are numerous factors that influence the ultimate success of vaginal delivery. Therefore, this study focuses specifically on exploring better methods for predicting cervical maturity and successful induction.

In this study, we used several machine learning methods to predict the time from induced labor to labor using clinical and ultrasound data and showed a promising cervical maturity evaluation method. Our goal is to establish a model that can accurately and objectively evaluate the cervical maturity and provide a more reliable decision-making basis for the clinical diagnosis of obstetrics and gynecology. Compared with the traditional Bishop scoring system based on clinical scoring by doctors, our cervical maturity prediction and evaluation method based on machine learning have more objective and accurate characteristics. When using ultrasound data, Bishop, and other clinical data, our method is significantly better than the traditional simple Bishop score system and achieves the best results. After removing the feature of Bishop score, the effect of machine learning model using only ultrasound data and obstetric clinical data decreased slightly, but there was also a prominent statistically significant difference compared with the traditional Bishop scoring method, whereas the RF method was not affected, after removing the feature of Bishop score. When dealing with the category feature of the method of induced labor and primiparity/multiparity, as the feature base is very low, we use one-hot coding to represent all the feature values, which is not enough to cause dimensional disaster and does not affect the ability of CatBoost to deal with category feature.

On the basis of the importance of several features in several machine learning methods, we preliminarily speculate that cervical length, Bishop score, angle, age, ILT, MT, and whether the use of amniotomy in the method of induced labor are more important features to predict the cervical maturity. This will also highlight on what doctors need to pay attention in clinical practice.

This is a preliminary study, and there are still some limitations in the current work. In this study, our purpose is to predict the time from induced labor to labor and to evaluate the cervical maturity, not to study the causal relationship between cervical maturity and pregnancy variables. In the future, we will collect more data and use more methods to calculate the importance of features to study the factors affecting cervical maturity. Bishop score includes five scoring dimensions: dilatation, effacement, station, consistency, and position. The experimental results show that Bishop score is significant in predicting the time from induced labor to labor, but the score is subjective. In the follow-up, we will further study which of the five dimensions of Bishop score plays an important role. At present, because of the development of cervical elasticity ultrasound technology, we can easily obtain the objective cervical elasticity data. In future, we can collect more cervical elasticity data, re-train the model, explore a cervical maturity evaluation method that completely depends on objective data, and abandon the influence of personal subjective factors on the prediction of the time from induced labor to labor.

## Conclusions

The machine learning method is a better predictor of cervical maturity than the traditional Bishop method. The prediction accuracy of machine learning model usually increases with the increase of training data. Improvement on the prediction model might be achieved when lager amount of data is obtained in future.

## Data Availability

All data generated or analysed during this study are available from the corresponding author on reasonable request.

## Abbreviations

ILT: Induction of labor time, induced labor time
MT: Measurement time
ILTLT: The time from induction of labor to labor
MTILT: Measurement time to induced labor time
RF: Random forest
MAE: Mean absolute error
NT: Nonstress Test
CART: Classification and Regression Tree
XGBoost: EXtreme Gradient Boosting
CatBoost: An implementation of Gradient Boosted Decision Trees
